# Echocardiographic Features of Cardiomyopathy in Emery-Dreifuss Muscular Dystrophy

**DOI:** 10.1155/2021/8812044

**Published:** 2021-02-04

**Authors:** Michał Marchel, Agnieszka Madej-Pilarczyk, Agata Tymińska, Roman Steckiewicz, Janusz Kochanowski, Julia Wysińska, Ewa Ostrowska, Paweł Balsam, Marcin Grabowski, Grzegorz Opolski

**Affiliations:** ^1^1st Department of Cardiology, Medical University of Warsaw, Warsaw, Poland; ^2^Department of Medical Genetics, The Children's Memorial Health Institute, Warsaw, Poland

## Abstract

**Background:**

Emery-Dreifuss muscular dystrophy (EDMD) is a very rare type of muscular dystrophy characterized by musculoskeletal abnormalities accompanied by cardiac defects. Two most common genetic subtypes are EDMD1 due to *EMD* and EDMD2 caused by LMNA gene mutations. The aim of the study was to characterize and compare the cardiac morphology and function in the two main genetic subgroups of EDMD with the use of echocardiography.

**Methods:**

41 patients with EDMD (29 EDMD1 and 12 EDMD2) as well as 25 healthy controls were enrolled in our study. Transthoracic echo with the use of a prescribed protocol was performed.

**Results:**

Highly statistically significant differences with regard to left ventricle (LV) volumes between the EDMD and the control group were found. 51% of EDMD patients had an enlarged left atrium and as many as 71% had an enlarged right atrium. The LV ejection fraction (LVEF) was significantly lower in EDMD patients than in the control group which corresponded also with a lower systolic velocity of the mitral annulus. 43% of EDMD patients had LVEF below the normal limit. Diastolic dysfunction was detected in 17% of EDMD patients. There were no significant differences between the two types of EDMD in terms of diameters and volumes of any chamber, as well as the systolic function of both left and right ventricles.

**Conclusions:**

A significant number of EDMD patients present LV dilatation and different degrees of systolic dysfunction. Dilatation of the atria dominates over ventricle dilatation. We did not present any significant differences between EDMD1 and EDMD2 in terms of the morphology and the function of the heart.

## 1. Introduction

Emery-Dreifuss muscular dystrophy (EDMD) is a very rare type of muscular dystrophy with a prevalence of 0.39 per 100 000 [1]. It is characterized by musculoskeletal abnormalities: early joint contractures, generalized muscle atrophy, and slowly progressing weakness accompanied by cardiac defects [2, 3]. Typical cardiac findings include conduction disturbances, supraventricular arrhythmias, cardiac chamber dilatation, and systolic dysfunction. EDMD patients are at high risk of sudden cardiac death. Many of them require cardiac pacing [4] or implanted cardioverter-defibrillator (ICD) [5]. There are several genetic subtypes of EDMD [1–7], with EMD (EDMD1) and LMNA (EDMD2) being the two most common mutated genes [6–11]. The type and extent of cardiac involvement may differ between the two main genetic types. Patients with LMNA mutation are at high risk of sudden death [12] because of ventricular arrhythmias and/or fast progressing cardiac failure in the course of cardiomyopathy, while EMD patients suffer more from supraventricular arrhythmias; moreover, atrial standstill is a typical phenomenon [13]. There are some data focusing on left ventricular (LV) systolic function [14, 15] and case series describing cardiac dysfunction [16, 17], but there is a lack of systematic assessment of the right ventricle as well as both atria. The aim of the study was to characterize and compare the cardiac morphology and function in the two main genetic subgroups of EDMD.

## 2. Materials and Methods

### 2.1. Study Design

41 patients with EDMD as well as 25 healthy age-matched controls were prospectively enrolled in our study. All patients were referred by a neurologist dedicated to neuromuscular patient outpatient cardiology clinic (1st Department of Cardiology, Medical University of Warsaw) at the time of EDMD diagnosis. All patients had genetically confirmed mutation in EMD (EDMD1) or LMNA (EDMD2) genes. The exclusion criterion was cardiovascular disease other than EDMD. Height and weight were recorded to calculate the body surface area. Written informed consent was obtained from all the subjects.

### 2.2. Neurologic Assessment

Skeletal muscle involvement has been arbitrarily divided into three groups according to the severity of symptoms and signs:

- Mild (weakness and wasting of humeroperoneal muscles, mild joint contractures in elbows/ankles, and mild spine rigidity; functionally: patient is able to climb stairs without a handrail and efficiently gets up from the squatting position).
- Moderate (generalized muscle atrophy and wasting, evident joint contractures in elbows/ankles, and spine rigidity; functionally: patient is not able to climb stairs or get up from the squatting position unassisted).
- Severe (generalized muscle atrophy and wasting, multiple joint contractures, and spine rigidity; functionally: physically disabled or wheelchair bounded and also patients with cerebral stroke complications).

### 2.3. Echocardiographic Assessment

Since the majority of EDMD patients require pacing at some point in the course of the disease and the acoustic window is of accepted quality in a vast majority of EDMD patients, echocardiography was chosen as the most sufficient method of cardiac function assessment. Transthoracic echo with the use of a prescribed protocol was performed. Diameters and volumes of the chambers as well as the systolic and diastolic function were measured in accordance with the guidelines [18–21]. End-diastolic (LVEDd) and end-systolic (LVESd) diameters of LV and the end-systolic diameter of the left atrium (LAd) (parasternal long-axis view) as well as end-diastolic (LVEDV) and end-systolic (LVESV) LV volumes and end-systolic left (LAV) and right (RAV) atrial volume from four- and two-chamber apical views were measured. All diameters and volumes were indexed to the body surface area. LV ejection fraction (LVEF) was measured according to the Simpson method. Tricuspid annular plane systolic excursion (TAPSE) was measured to estimate the right ventricular systolic function. Maximal velocity of early mitral inflow (E, pulse wave Doppler) and systolic (s') and diastolic (e'; a') velocities of septal and lateral basal regions using pulse wave tissue Doppler were measured. Tricuspid regurgitation maximal velocity and propagation velocity of mitral inflow with the use of color Doppler and the m-mode technique were measured as well. Images were obtained with the S5-1 5 Mhz transducer (Philips) with IE-33 and EPIC 7 ultrasound scanner (Philips) with synchronous electrocardiogram recording. The measurements were performed with the use of a dedicated software package (Xcelera, Q-Station, Philips Healthcare, Andover, MA). The echocardiographist was blinded to the genetic results. The plasma levels of cardiac biomarkers (NT-proBNP, Roche Diagnostics GmbH, Mannheim, Germany; NT-proANP, Biomedica Medizinprodukte^®^ GmbH, Wien, Austria) were determined.

### 2.4. Statistical Analysis

For an intergroup comparison, we used Fisher's exact test for categorical variables and the Mann-Whitney U test for continuous and ordinal variables. Data are presented as median value and interquartile range (IQR). Pearson's and Spearman's correlation coefficients were used for parametric and nonparametric variables, respectively. For all tests, *p* value below 0.05 was considered significant. All tests were two-tailed. Statistical analyses were performed using SPSS version 22 software.

## 3. Results and Discussion

### 3.1. Patient Characteristics

The study involved 29 patients (median age 43 (31–55 years)) with EDMD1 (12 sporadic and 17 familial cases) and 12 with EDMD2 (7 sporadic and 5 familial cases). There were no statistically significant differences between the study and control groups in terms of age and body mass index. Natriuretic peptide levels were significantly higher in EDMD compared to the control group (NT-proBNP: 74.6 (49.2–168.6) versus 31.0 (15.6–66.2) pg/ml, *p* < 0.001, and NT-proANP: 1.0 (0.8–1.9) versus 0.8 (0.6–0.8) pg/ml, *p* < 0.001).  Table 1 contains the baseline characteristics of the study groups.

### 3.2. Evaluation of Cardiac Chamber Diameters and Volumes

**Figure 1:**
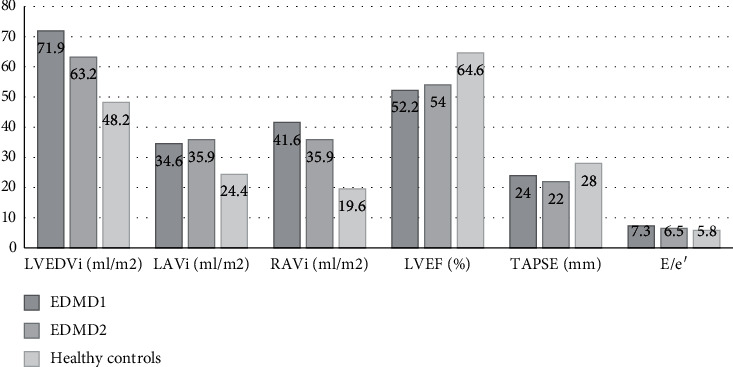
Cardiac chamber volumes and systolic and diastolic parameters of EDMD1, EDMD2, and healthy controls. E/e': mitral early peak velocity/annular velocity; LAVi: left atrial volume index; LVEF: left ventricular ejection fraction; LVEDVi: left ventricular end-diastolic volume index; RAVi: right atrial volume index.

Highly statistically significant differences with regard to LV end-diastolic and end-systolic indexed volumes between the EDMD and the control group were found. The same was observed for LV dimensions (Table 2). As many as 41% of EDMD patients had enlarged LV in relation to normal values [19]. The right ventricular dimension did not differ between EDMD and healthy controls. The right ventricle was enlarged in only 12% of EDMD patients. Moreover, diameters and volumes of the left and right atria were higher in the EDMD group. The median RAVi was two times higher in EDMD patients than in controls. 51% of EDMD patients had an enlarged left atrium and as many as 71% had an enlarged right atrium (Figure 1 and Table 2).

### 3.3. Evaluation of Systolic and Diastolic Function

The LVEF was significantly lower in EDMD patients than in the control group which corresponded also with a lower systolic velocity of the mitral annulus (s') measured in tissue Doppler (Table 3). 43% of EDMD patients had LVEF below the normal limit (LVEF < 52% for males and <54% for females). Significantly reduced ejection fraction (LVEF < 40%) was documented in 12% (5/41) of all patients (2 EDMD1 and 3 EDMD2), and the so-called midrange LVEF (40–49%) was present in 20% (8/41) of patients. (Table 4)

Although the right ventricle was not enlarged in EDMD (Table 2), the right ventricular systolic function estimated by the measurement of TAPSE was significantly lower in the EDMD grroup than in the control group (Table 3). Significant right ventricle systolic dysfunction (TAPSE < 17 mm) was present in 10% (4/41) of patients.

In terms of diastolic dysfunction, significantly higher E/e' and right ventricle systolic pressure, in addition to lower propagation velocity, were documented (Table 3). Diastolic dysfunction based on guidelines criteria [22] was detected in 17% (7/42) of EDMD patients (4 EDMD1 and 3 EDMD2), although no significant hypertrophy of the left or the right ventricle was noticed. We found correlations between diastolic parameters and NT-proBNP (E/e' and NT-proBNP; Rho 0,63, *p*=0,001) in the EDMD patients.

There were no significant differences between the two types of EDMD in terms of diameters and volumes of any chamber. Similarly, the systolic function of both left and right ventricles did not differ. Although isovolumetric relaxation time was significantly shorter in EDMD2 patients, no other diastolic parameters differed. Moreover, A and a' velocities corresponding with the mechanical function of the left atrium responsible for late filling of the LV did not differ between the subgroups. Echocardiographic data are presented in Tables 2 and 3.

### 3.4. Correlation with Timing of Assessment and Peripheral Muscle Involvement

Patients differed in terms of the time of their evaluations. According to the protocol, the echocardiogram was performed when EDMD diagnosis was established, although its timing was not parallel with the onset of muscle symptoms (median of 5 (0–10) years from the beginning of the first symptoms to EDMD diagnosis). Bearing in mind that EDMD is a progressive disease, we examined if there was any correlation between the duration of peripheral muscle symptoms and echocardiographic parameters. We found that the later the echocardiogram was performed since the beginning of the symptoms, the higher the NT-proBNP is (*r* = 0.47; *p*=0.01). There was also a close to significant correlation between lower LVEF (*r* = −0.36, *p*=0.056) and progression of neurologic symptoms. No other echocardiographic parameters correlated with the duration of EDMD.

Also, we investigated the occurrence of any significant differences in echocardiographic parameters between EDMD patients with different degrees of the severity of peripheral muscle involvement. Among different parameters indexed, RAV volume was the only one which was significantly higher in patients with more severe dystrophy (23.1 (13.5–27.6) versus 44.3 (30.1–67.6) ml/m2, *p* < 0.02 for mild versus more severe symptoms, resp.).

## 4. Discussion

The study assessed the cardiac morphology and function in a group of patients with EDMD. EDMD is one of those very rare muscular dystrophies, in which peripheral muscle involvement may be benign in some patients; however severe disability due to skeletal muscle disease may also occur, especially in EDMD2. The main risk for the patients depends on cardiac abnormalities [23]. Our knowledge about cardiomyopathy in the course of EDMD is supported by few papers, case reports [24], and case series reports [17, 25]. The main message regarding cardiomyopathy from these studies showed that, in contrast to Duchenne muscular dystrophy (DMD) or Becker muscular dystrophy (BMD), typical dilated cardiomyopathy (DCM) is seen only in a percentage of patients. In some of them, conduction disturbances/heart arrhythmias are accompanied by atrial dilatation, especially at the early stage of the disease (frequently observed in EDMD1), while in others, systolic dysfunction was found (more often in EDMD2). Pattern of skeletal muscle involvement does not correlate with the extent of cardiac disease; however, the sequence of muscle and cardiac symptoms is easier to predict in EDMD1, where muscle weakness/wasting and joint contractures typically are seen before heart disease. In EDMD2, timing and severity of muscle and cardiac involvement are more variable, as there are patients with mild skeletal muscle symptoms and severe course of heart disease [7, 13]. Penetration of LMNA mutations as for cardiac involvement is almost complete, so one may expect that all patients with EDMD2 will develop heart dysfunction at some stage of their disease [26]. Sanna et al. [15] presented 10 patients with EDMD2 in whom four developed LV systolic dysfunction. Three out of these four patients presented nonsignificant dilatation of the LV. In Boriani's series of 18 patients [27], only two EDMD1 patients developed significant systolic dysfunction, while one patient with EDMD2 developed end-stage heart failure with reduced LVEF and had a heart transplant. Bonne et al. [11] presented a cohort of EDMD2 patients. In 35 cases, echocardiography was performed, and in 9/35 patients, ventricular dysfunction was present. No more details about the LV volumes or LVEF were provided. In a paper by Smith et al. [28], eight patients with EDMD2 were tested with echocardiography and cardiac magnetic resonance (CMR). No significant differences in cardiac morphology and function were presented although the mean age of the studied population was only 18.5 years. No myocardial fibrosis in CMR was detected in the early stages of LMNA cardiomyopathy. Draminska [14] presented echocardiographic data on LV diameters and LVEF. However, LVEF was calculated using the Teichholz method, which is not recommended any more [19, 21]. 3/27 patients had an enlarged LV; the mean end-diastolic indexed diameters were higher than in controls. The data regarding volumes and atria were not provided. In many previously published papers describing cardiac involvement, both ventricular and atrial functions were not reported in detail [29, 30]. In our cohort, patients were older at the time of diagnosis of cardiac disease. The median age of our population was 43 years. In the other studies, the patients were usually in their second or third decade of life [14, 15].

In this study, we described cardiac morphology and function in detail in the big cohort of Polish patients with EDMD1 and EDMD2. We found higher diameters and volumes of the LV in comparison with the controls. As many as 41% of EDMD patients had LVEDV above normal values. The differences were highly significant. Although the median value of LVEF was 52.5%, which is near the lower limit of normal values, 32% of patients presented with truly depressed LVEF (<50%). Among those with cardiac remodeling (increased LVESVi), still 17% (7/41) had preserved LVEF. This finding may be supportive of the thesis that, in EDMD, a decrease of the LV systolic function is not completely parallel to the enlargement of cardiac chambers [31]. Much less, only 12% (5/41) of EDMD presented right ventricle enlargement. 4/5 from this group have concomitant LV dilatation as well. Although the right ventricle was not enlarged, its systolic function was still slightly decreased compared to the control group. Buckley et al. [17] postulated that, in the cardiomyopathy of EDMD patients, not only atria but also right heart involvement predominates, which was not confirmed in our study.

In the group with heart failure, not only ventricle enlargement and decreased systolic function but also increased volumes of the left and right atria have some prognostic impact [32]. In this cohort of EDMD patients, more than two-thirds presented right atrium enlargement. Considering the absence of right ventricular dysfunction, this may suggest early atrial muscle involvement. This is consistent with the predominance of supraventricular arrhythmias in the early stages of cardiomyopathy in the course of EDMD [15, 27]. Also, the volumes of right atria were impressive, with 20% of patients having RAV above 100 ml in absolute numbers. The first report of marked right atrium dilatation was published by Buckley et al. in their case series of three patients from one family [17]. In a paper by Carboni et al. describing a family with EDMD1, variable biatrial dilatation was described [25] with no other details. In a paper by Boriani et al. [27], the right atrial dilatation was present in 67% (12/18) and the left atrial dilatation was present in 39% (7/18), which is similar to our results.

There is no data on diastolic dysfunction in EDMD. Considering the increased risk of stroke in this type of muscular dystrophy and the correlation between impaired diastolic parameters and increased risk of thromboembolic complications in patients with atrial fibrillation and preserved LVEF [33], the assessment of diastolic function is of special interest. Diastolic dysfunction of the LV was present in 17% (7/41) of the patients, which lead to elevated filling pressures in patients without systolic dysfunction. It may also be a sign of increased stiffness of the ventricle due to the fibrosis of the myocardium [34]. Interestingly, the higher filling pressures were concordant with increased estimated pressure in the pulmonary artery and correlated significantly with NT-proBNP (rho 0.62, *p* < 0.001), which has a documented predictive value for ventricular arrhythmias in DCM in laminopathy [30].

Differences in cardiac presentation, including the severity of LV enlargement and systolic dysfunction, were postulated [7, 35]. LMNA is one of the most common DCM-causing genes in patients with no peripheral muscle involvement. In the general population of DCM patients, LMNA mutations are found in 5% of the nonfamilial cases and in 5–10% of the familial ones. If an atrioventricular block is present together with DCM, the probability of Lamin A/C mutation rises to around 33% [36]. In contrast, EMD mutation is a rare phenomenon in pure DCM [35, 37]. One could expect more severe cardiomyopathy in EDMD2 than in EDMD1. However, our results do not confirm this thesis. The extent of both ventricles and atria involvement echocardiographically examined was similar in EDMD of both genetic backgrounds.

We also determined whether there were any significant differences in echocardiographic parameters between EDMD patients with different severities of peripheral muscle involvement. Among various parameters, indexed RAV was the only measure that was significantly higher in patients with more severe dystrophy (23.1 (13.5–27.6) versus 44.3 (30.1–67.6) ml/m2, *p* < 0.02, for mild versus more severe symptoms, resp.).

## 5. Conclusions

The picture of cardiomyopathy in the course of EDMD is different from other X-linked muscular dystrophies, in particular dystrophinopathies (DMD and BMD), where typical dilated cardiomyopathy is present. A significant number of EDMD patients present LV dilatation and different degrees of systolic dysfunction, although mild systolic dysfunction is the most common. Diastolic dysfunction of the LV was present in a significant number of patients as well. Right ventricular dilatation and dysfunction are much less common. More than half of the patients have not only left but also right atrium enlargement. In EDMD, dilatation of the atria dominates over ventricle dilatation. We did not present any significant differences between EDMD1 and EDMD2 in terms of the morphology and the function of the heart.

## Figures and Tables

**Table 1:** Baseline characteristics of the study population.

	EDMD patients, *n* = 41	Healthy controls, *n* = 25	*p* value
Age (years)	43 (31–55)	37 (36–44)	0.17
Female (%)	36.5 15/41	48.0; 12/25	0.81
BMI (kg/m2)	20.8 (19.3–25.3)	22.6 (20.4–24.1)	0.37
BSA (m2)	1.72 (1.55–1.83)	1.77 (1.62–1.93)	0.10
EMD (sporadic/familial)	12/17		
LMNA (sporadic/familial)	7/5		
Muscular involvement (mild/moderate/severe)	18/19/4		
NT-proBNP (pg/ml)	74.6 (49.2–168.6)	31.0 (15.6–66.2)	<0.001
NT-proANP (pg/ml)	1.0 (0.8–1.9)	0.8 (0.6–0.8)	<0.001

BMI: body mass index; BSA: body surface area; EMD: mutation in EMD gen; LMNA: mutation in LMNA gene; NTpro-BNP: N-terminal prohormone B-type natriuretic peptide; NT-proANP: N-terminal prohormone A-type natriuretic peptide.

**Table 2:** Cardiac chamber diameters and volumes of EDMD patients and healthy controls.

	EDMD1 patients, *n* = 29	EDMD2 patients, *n* = 12	Healthy controls, *n* = 25	*p* value, EDMD1 versus EDMD2	*p* value, EDMD versus control
LVEDdi (mm/m2)	29.3 (26.8–32.0)	32.0 (29.9–33.2)	25.5 (23.5–27.6)	0.26	<0.001
LVEDVi (ml/m2)	71.9 (53.7–92.4)	63.2 (56.2–86.2)	48.2 (43.0–51.9)	0.83	<0.001
LVESdi (mm/m2)	19.1 (15.4–23.1)	20.3 (16.6–22.1)	15.7 (14.3–17.9)	0.70	<0.001
LVESVi (ml/m2)	35.1 (24.0–45.6)	30.8 (22.9–48.4)	16.6 (14.1–18.9)	0.90	<0.001
RVdi (mm/m2)	12.8 (11.3–15.5)	14.4 (12.4–18.1)	12.9 (12.5–14.3)	0.18	0.37
LAdi (mm/m2)	20.7 (19.2–22.7)	23.4 (20.1–24.6)	18.4 (17.8–20.0)	0.07	<0.001
LAVi (ml/m2)	34.6 (26.9–39.6)	35.9 (24.9–39.2)	24.4 (20.7–26.4)	0.81	<0.001
RAVi (ml/m2)	41.6 (28.6–76.7)	35.9 (28.5–47.9)	19.6 (17.8–22.9)	0.53	<0.001

LAdi: left atrium dimension index; LAVi: left atrial volume index; LVEDdi: left ventricular end-diastolic dimension index; LVEDVi: left ventricular end-diastolic volume index; LVESdi: left ventricular end-systolic dimension index; LVESVi: left ventricular end-systolic volume index; RAVi: right atrial volume index; RVdi: right ventricular dimension index.

**Table 3:** Evaluation of systolic and diastolic function in EDMD and healthy controls.

	EDMD1 patients, *n* = 29	EDMD2 patients, *n* = 12	Healthy controls, *n* = 25	*p* value, EDMD1 versus EDMD2	*p* value, EDMD versus control
LVEF (%)	52.2 (46.4–58.7)	54.0 (43.0–59.0)	64.6 (62.1–67.3)	0.94	<0.001
s' (cm/s)	8.0 (7.0–10.1)	8.3 (6.8–9.1)	8.7 (8.4–9.1)	0.90	0.02
TAPSE (mm)	24.0 (18.5–25.5)	22.0 (18.3–24.0)	28.0 (24.0–30.0)	0.56	<0.001
TRPG (mmHg)	20.0 (16.5–31.0)	19.5 (0.0–30.0)	10.0 (10.0–16.0)	0.62	<0.0001
e' (cm/s)	9.8 (8.1–11.3)	8.0 (6.7–10.5)	11.6 (11.0–12.5)	0.27	<0.001
a' (cm/s)	6.7 (5.9–7.4)	6.5 (6.2–7.6)	8.4 (7.6–9.7)	1.00	1.00
E/e'	7.3 (5.4–10.2)	6.5 (5.8–13.1)	5.8 (5.5–6.4)	0.74	0.01
LAVi (ml/m2)	34.6 (26.9–39.6)	35.9 (24.9–39.2)	24.4 (20.7–26.4)	0.81	<0.001
Vprop (cm/s)	61.8 (50.6–79.4)	68.6 (40.9–73.8)	71.0 (62.9–90.4)	0.86	0.03
IVRT (ms)	85.0 (72.5–95.5)	69.0 (61.0–79.5)	81.0 (66.5–91.5)	0.02	0.84

a': late diastolic annulus velocity; e': early diastolic annulus velocity; E/e': mitral early peak velocity/annular velocity; IVRT: isovolumetric relaxation time; LVEF: left ventricular ejection fraction; LAVi: left atrial volume index; s': systolic annulus velocity; TAPSE: tricuspid annular plane systolic excursion; TRPG: tricuspid regurgitation pressure gradient; Vprop: inflow propagation velocity.

**Table 4:** Percentage of EDMD patients meeting the following criteria.

	EDMD1	EDMD2	*p* value
LVEDV >150/106 ml (M/F) (%)	41.4; 12/29	41.7; 5/12	1.00
LVEDVi > 74/61 ml/m2 (M/F) (%)	44.8; 13/29	41.7; 5/12	1.00
LAVi > 34 ml/m2 (%)	48.3; 14/29	58.3; 7/12	0.74
RAVi > 32/27 ml/m2 (M/F) (%)	69.0; 20/29	75.0; 9/12	1.00
RV > 30 mm (%)	6.9; 2/29	25.0; 3/12	0.14
LVEF < 52/54 % (M/F) (%)	41.4; 12/29	41.7; 5/12	1.00
TAPSE < 17 mm (%)	6.9; 2/29	16.7; 2/12	0.57
E/e' > 14 (%)	16.0; 4/25	16.7; 2/12	1.00
Vprop < 45 cm/s (%)	12.5; 3/24	33.3; 4/12	0.19

E/e': mitral early peak velocity/annular velocity; LAVi: left atrial volume index; LVEDV: left ventricular end-diastolic volume; LVEDVi: left ventricular end-diastolic volume index; LVEF: left ventricular ejection fraction; RAVi: right atrial volume index; RV: right ventricle; TAPSE: tricuspid annular plane systolic excursion; Vprop: inflow propagation velocity.

## Data Availability

The echocardiographic data used to support the findings of this study are included within the article. Some of the genetic data (i.e., detailed mutations) may be available from the corresponding author upon request although some of them may be restricted in order to protect patient privacy.
